# Flavonoids from *Praxelis clematidea* R.M. King and Robinson Modulate Bacterial Drug Resistance

**DOI:** 10.3390/molecules16064828

**Published:** 2011-06-10

**Authors:** Gabriela Lemos de Azevedo Maia, Vivyanne dos Santos Falcão-Silva, Pedro Gregório Vieira Aquino, João Xavier de Araújo-Júnior, Josean Fechine Tavares, Marcelo Sobral da Silva, Luis Cezar Rodrigues, José Pinto de Siqueira-Júnior, José Maria Barbosa-Filho

**Affiliations:** 1 Laboratory of Pharmaceutical Technology, Federal University of Paraíba, João Pessoa, PB 58051, Brazil; Email: josean@ltf.ufpb.br (J.F.T.); marcelosobral@ltf.ufpb.br (M.S.S.); lcezar@ltf.ufpb.br (L.C.R.); 2 Academic Collegiate of Pharmaceutical Sciences, Federal University of Sao Francisco Valley, Petrolina, PE 56304, Brazil; 3 Laboratory of Genetics of Microorganisms, Department of Molecular Biology, Federal University of Paraíba, João Pessoa, PB 58059, Brazil; Email: vivyannefalcao@yahoo.com.br (V.S.F.-S.); jpsiq@uol.com.br (J.P.S.-J.); 4 Laboratory of Research on Natural Resources, Institute of Chemistry and Biotechnology, Federal University of Alagoas, Maceió, AL 57072, Brazil; Email: pgvaquino@hotmail.com (P.G.V.A.); joaoxjr@yahoo.com.br (J.X.A.-J.)

**Keywords:** *Praxelis clematidea*, Asteraceae, flavonoids, antibacterial activity, bacterial resistance, efflux pump

## Abstract

Chemical studies of *Praxelis clematidea* R.M. King & Robinson resulted in the isolation of six flavones: Apigenine, genkwanine, 7,4’-dimethylapigenin, trimethylapigenin, cirsimaritin and tetramethylscutellarein, which were tested for their toxicity against *Staphylococcus aureus* SA-1199B, a strain possessing the NorA efflux pump. Efflux pumps are integral proteins of the bacterial membrane and are recognized as one of the main causes of bacterial drug resistance, since they expel antibiotics from the cell. The inhibition of this transporter is one form of modulating bacterial resistance to antimicrobial drugs. The flavones tested did not show any significant antibacterial activity against the *Staphylococcus aureus* strain used, but were able to modulate bacterial drug resistance. This property might be related to the degree of lipophilicity of the flavones conferred by the methoxyl groups, since 4’,5,6,7 tetramethoxyflavone the most methoxylated compound, reduced the minimal inhibitory concentration of the drug 16-fold.

## 1. Introduction

The family Asteraceae is the largest Angiosperm group, consisting of approximately 23,000 species distributed in 1,535 genera. It has a cosmopolitan distribution, and found on all the continents except Antarctica. South America is home to about 20% of the existing genera. In Brazil, there are approximately 180 genera and 3,000 species distributed throughout the country.

Plants from this family have been extensively studied for their chemical composition and biological activity and some have led to the development of new drugs and insecticides [1,2,3,4,5,6,7,8,9,10,11,12,13].

*Praxelis clematidea* R.M. King & Robinson belongs to the Eupatorieae tribe of the family Asteraceae, and consists of 2,400 species distributed in 170 genera [14]. The species has the following synonyms: *Eupatorium clematideum* Griseb. and *Eupatorium urtifolium* var. *clematideum* (Griseb.) Hieron ex. Kuntze.

It is a perennial weed native to South America and distributed throughout Bolivia, Peru and Argentina. In Brazil, it is found mainly in the states of Bahia, Alagoas, Pernambuco, Paraiba, Amazonas and Mato Grosso [15]. In phytochemical studies, Bolhmann and coworkers [16] isolated *N*-(acetoxy)-jasmonoylphenylalanine-methyl-ester. Gas chromatographic analysis showed the presence of sesquiterpenes and monoterpenes in the essential oil extracted from *P. clematidae*, which also showed growth inhibitory effect on two plant species, *Lactuca sativa* and *Brassica campestris*, and on fungal colonies of *Fusarium oxysporum* and *Phytopthora capsici* [17]. A pharmacological study conducted with the aerial parts of this species demonstrated significant gastroprotective activity against gastric ulcers induced in animals with ethanol, stress, and a non-steroidal antiinflammatory [18].

The studies on *Praxelis clematidea* don’t report the presence of flavonoids, although scientific studies conducted on the family Asteraceae have identified flavonoids as important chemotaxonomic markers of this family [19]. Based on this information, we started with the aerial parts of *Praxelis clematidea* to isolate compounds belonging to this secondary metabolite class. This class is increasingly becoming an object of investigation, and many studies have isolated and identified flavonoids that possess antifungal, antiviral and antibacterial activities. In addition, various studies have demonstrated synergy between active flavonoids, and between flavonoids and conventional chemotherapeutic agents [20,21].

The ever increasing bacterial resistance to antibiotics is a serious problem for public health that affects most current antibacterial agents. Efflux pumps are integral proteins of the bacterial membrane and are recognized as one of the major sources of bacterial resistance since they extrude antibiotics from the cell [22,23].

Modulators of antibiotic drug resistance are compounds that potentiate antibiotic activity against resistant strains. Some of these agents act as efflux pump inhibitors (EPIs) [24,25]. Plants provide a rich source of EPIs and several compounds have been identified as potent inhibitors [26,27,28].

The aim of the present work was to isolate and characterize the structure of flavonoids from *Praxelis clematidea* and study their activity as modulators of drug resistance in *Staphylococcus aureus* SA-1199B.

Some methoxylated flavonoids that potentiate the activity of antimicrobial drugs have already been described [29,30,31,32]. However, as far as we know, none of the flavonoids presented here has been previously evaluated. The results add new scientific evidence that flavonoids modulate antibiotic resistance, probably by efflux pump inhibition.

## 2. Results and Discussion

**Figure 1:**
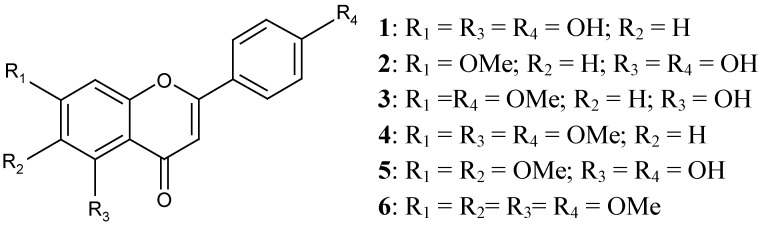
Isolated flavonoids from *Praxelis clematidea*.

The structural identification of the compounds (Figure 1) was carried out based on the analysis of the spectral data and by comparison with the literature [33,34]. The compounds were: (1) apigenine (4’,5,7-trihydroxyflavone), (2) genkwanin (4’,5-dihydroxy-7-methoxyflavone), (3) 7,4’-dimethylapigenin (5-hydroxy-4’,7-dimethoxyflavone), (4) trimethylapigenin (4’,5,7-trimethoxyflavone), (5) cirsimaritin (4’,5,-dihydroxy-6,7-dimethoxyflavone) and (6) tetramethylscutellarein (4’,5,6,7-tetramethoxyflavone).

Methoxylated flavones showed no antibacterial activity at 256 μg/mL against the tested strain of *S. aureus*. When the compounds were added to the growth medium at 64 µg/mL (1/4 MIC), a reduction in the MIC of at least two-fold (and up to 16-fold) was observed for norfloxacin and ethidium bromide (Table 1). All experiments were carried out at least twice with consistent results.

**Table 1:** Minimum inhibitory concentrations (MICs) of antibiotics and ethidium bromide against *Staphylococcus aureus* strain SA-1199B, in the absence and presence of flavones.

Flavones	MIC (µg/mL)		
	Norfloxacin	Ethidium bromide	Pefloxacin
None	128	32	16
**1**	128	32	16
**2**	64 (2×) ^a^	16 (2×)	16
**3**	64 (2×)	16 (2×)	16
**4**	16 (8×)	8 (4×)	16
**5**	32 (4×)	8 (4×)	16
**6**	8 (16×)	2 (16×)	16

^a^ Fold reduction in MIC.

Methoxylated flavones modulate drug activity by reducing the concentration needed to inhibit the growth of the drug-resistant (effluxing) bacteria. This activity may be related to flavanoid lipophilicity due to the presence of methoxyl groups. Lipophilicity is a common feature of several efflux pump inhibitors and may be a key factor for inhibition in Gram-positive bacteria [28].

Ethidium bromide is a well-known substrate for the NorA efflux protein, and active efflux is the only known mechanism of resistance to this DNA-intercalating dye [35]. Therefore, the use of ethidium bromide against the strain SA-1199B was used to demonstrate that the methoxylated flavones evaluated here modulated norfloxacin resistance by efflux pump inhibition.

Pefloxacin, a hydrophobic quinolone, is a poor substrate of the NorA efflux pump, and it was used as a negative control [25]. Reductions in MICs of norfloxacin and ethidium bromide when combined with chlorpromazine or trifluoperazine were also observed (data not shown), and the results were consistent with those reported by Kaatz *et al.* [24] and by Falcão-Silva *et al.* [32]; both phenothiazines were used as positive (internal) control.

The results can be explained by the increasing lipophilicity in the compounds. An analysis of log P values for the compounds (calculated with ChemDraw Ultra 10.0, Cambridge Software) revealed the following order of lipophilicity: **1** (log P 1.9) < **5** (log P 2.04) < **2** (log P 2.17) < **3** (log P 2.43) < **6** (log P 2.57) < **4** (log P 2.69). This order explains, in part, the following order of activity: Nor: **1** < **2**/**3** < **5** < **4** < **6** and EB: **1** < **2**/**3** < **5**/**4** <**6**.

The importance of the presence of a methoxyl in the 4’ position [29] was also observed, as the activity of compounds **3** and **4** was higher than that of compounds **1** and **2**, as well as the activity of compound **6** in relation to that of compound **5**. Another important factor was the total number of methoxyls in the flavonoids, in general, compounds **5** and **6** were more active than the other compounds, which is evident when we observe that compound **5** has a greater lipophilicity only compared to compound **1** and does not show a methoxyl in the 4’ position, even though it is more active than compounds **1**, **2** and **3**.

## 3. Experimental

### 3.1. General

The NMR spectra were obtained with a Mercury-Varian spectrometer at 200 MHz (^1^H) and 50 MHz (^13^C) and a VARIAN System model operating at 500 MHz (^1^H) and 125 MHz (^13^C). The solvents used were CDCl_3_, CD_3_OD and DMSO-*d_6_*, whose characteristic peaks in ^1^H and ^13^C-NMR are used to adjust the frequency scale. For column chromatography, silica gel 60 (70-230 Mesh) from Merck was utilized as the stationary phase. PF_254_ silica gel from Merck was used for the analytical (ATLC) and preparative (PTLC) thin-layer chromatography. The studied substances were identified by using ultraviolet radiation at wavelengths of 254 and 366 nm and by impregnation of plates in glass containers saturated with iodine vapor.

### 3.2. Plant Material

The aerial parts of *Praxelis clematidea* R.M. King & Robinson were collected in Lagoa do Paturi, a municipality of Santa Rita, in the state of Paraiba (Brazil), in May 2008. The identification of the botanical material was performed by Prof. Dr. Maria de Fatima Agra, Botany Sector, Laboratory of Pharmaceutical Technology/UFPB “Professor Delby Fernandes de Medeiros”. Exsiccates of the plant are deposited in the Prof. Lauro Pires Xavier (JPB) Herbarium, Paraiba Federal University, under the code M. F. Agra *et al*. 6894 (JPB).

### 3.3. Extraction and Isolation

The dried and pulverized plant material (aerial parts, 10 kg) was submitted to exhaustive maceration utilizing ethanol as the extraction solvent (3 × 10 L, every 72 h). The ethanolic solution obtained was concentrated in a rotary evaporator under reduced pressure, resulting in a crude ethanolic extract (600 g). This was partitioned with hexane, chloroform and ethyl acetate. The chloroform phase (120.96 g) was submitted to adsorption column chromatography (CC) using silica gel as the stationary phase and chloroform and methanol as the mobile phase, both as pure or as binary mixtures of increasing polarity, resulting in 188 fractions. These samples were analyzed by ATLC, and after examination with under UV light and iodine vapor, they were classified according to their Rf values into 25 groups. The sub-fractions 10–18 and 64–70 appeared as yellow solids and were identified as compounds **3** and **5**. The subgroup 23–24 was submitted to PTLC, utilizing chloroform and methanol (97:3) and furnishing compounds **6**, **2** and **4**. The subgroup 77–80 was submitted to PTLC, using chloroform and methanol (95:5) and supplying compound **1**.

### 3.4. Bacterial Strains

The *S. aureus* strain used, SA-1199B, over expresses the norA gene encoding the NorA efflux protein which extrudes hydrophilic fluoroquinolones and other drugs such as DNA-intercalating dyes [24,36]. The strain, kindly provided by Professor Simon Gibbons (University of London), was maintained on blood agar base slants (Laboratorios Difco Ltda., Brazil), and prior to use, the cells were grown overnight at 37 °C in brain heart infusion broth (BHI–Laboratorios Difco Ltda., Brazil).

### 3.5. Antibiotics and Chemicals

Norfloxacin, pefloxacin and ethidium bromide were obtained from Sigma Aldrich Co. Ltd. (USA). The stock solutions of the flavones were prepared in DMSO, and the highest concentration remaining after dilution in broth (4%) caused no inhibition of bacterial growth.

### 3.6. Drug Susceptibility Testing and Modulation Assay

The minimum inhibitory concentrations (MICs) of the antibiotics and flavonoids were determined in BHI by micro-dilution assay, using a suspension of ca. 105 CFU/mL and a drug concentration range from 256 to 0.5 μg/mL (twofold serial dilutions). MIC is defined as the lowest concentration at which no growth is observed. A solution of resazurin (0.01% w/v in sterile distilled water) was used to detect bacterial growth by color change from blue to pink. For the evaluation of flavones as modulators of drug resistance, the “modulation assay” was used, a method that has been widely applied to identify potential EPIs [29], *i.e.*, the MICs of antibiotics were determined in the presence of the flavones (in the BHI) at a sub-inhibitory concentration.

### 3.7. Log P Estimation

The structures were drawn utilizing the ChemDraw Ultra® 10.0 program (CambridgeSoft, 1986–2005), which also estimate their log P values.

## 4. Conclusions

Six flavones were isolated from *Praxelis clematidea* and identified through ^1^H and ^13^C-NMR data. Assays were carried out with these compounds against a strain of *Staphylococcus aureus* SA-1199B, with NorA efflux pump activity, demonstrating that the highest methoxylated flavones showed the highest efflux pump inhibition, or modulating of bacterial resistance. Inhibition of the bacterial transporter is related to the lipophilicity of the compound and might confer selectivity when used with antimicrobials.
